# Amine-Impregnated Dendritic Mesoporous Silica for the Adsorption of Formaldehyde

**DOI:** 10.3390/mi15010030

**Published:** 2023-12-22

**Authors:** Ji Myeong Lee, Misun Kang, June-Seo Kim, Jae Young Bae

**Affiliations:** 1 Department of Chemistry, Keimyung University, Daegu 42601, Republic of Korea; 1mounteye1@gmail.com (J.M.L.); misun.kang@gmail.com (M.K.); 2 Division of Nanotechnology, Daegu Gyeongbuk Institute of Science & Technology (DGIST), Daegu 42988, Republic of Korea

**Keywords:** mesoporous silica, dendritic mesoporous silica, hierarchical mesoporous silica, amine impregnation method, formaldehyde adsorption, TEPA, 2,4-DNPH, formaldehyde-2,4-DNPH

## Abstract

To adsorb and remove formaldehyde, which is a harmful volatile organic chemical (VOC) detected indoors, an alkylamine was introduced into the substrate as a formaldehyde adsorbent. In this study, Tetraethylenepentaamine (TEPA) was introduced into the mesoporous silica using the amine impregnation method. Since the impregnated alkylamine can block the pores of the silica substrate, the pore size and pore volume are very important factors for its use as a substrate for an adsorbent. Focusing on the substrate’s pore properties, Santa Barbara Amorphous-15 (SBA-15) was chosen as a conventional one-dimensional pore-structured mesoporous silica, and dendritic mesoporous silica (DMS) as a three-dimensional pore-structured mesoporous silica. To 1 g each of silica substrate DMS and SBA-15, 0, 0.5, 1.5, and 2.5 g of TEPA were introduced. A fixed concentration and amount of formaldehyde gas was flowed through the adsorbent and then the adsorbent was changed to the 2,4-Dinitrophenylhydrazine (2,4-DNPH) cartridge to adsorb the remaining formaldehyde. According to the methods recommended by the World Health Organization (WHO) and National Institute for Occupational Safety & Health (NIOSH), the formaldehyde captured by 2,4-DNPH was analyzed using high-performance liquid chromatography (HPLC). A comparison of DMS and SBA-15 in the amine impregnation method shows that not only surface area, but also large pore size and high pore volume, contribute to the formaldehyde adsorption ability.

## 1. Introduction

Formaldehyde (H_2_-C=O, HCHO; CAS 50-00-0), which was discovered by Russian chemist Butlerov, has no scent or color, and is better known as formalin in an aqueous state, is one of the carbonyl compounds. It is not only found in industrial facilities, but also easily found in natural atmospheric environments as it comes from the decomposition or combustion of biomass. Recently, formaldehyde detected indoors has become a problem and the products made and used by humans, especially those made with woodworking, are the main indirect sources of indoor formaldehyde. Short-term exposure to indoor formaldehyde irritates mucous membranes such as the upper respiratory system and eyeball. It also causes asthma, allergies, and eczema, and long-term exposure leads to cancer. Therefore, to reduce the formaldehyde of indoor air and prevent post-damage, many countries have devised various regulations and guidelines, such as limiting the detection limit of formaldehyde from indoor furniture. These formaldehydes in our life are mainly divided into formaldehyde dissolved in drinking water and formaldehyde of indoor air. In the case of indoor formaldehyde, reducing the formaldehyde concentration of indoor air is a problem. Fortunately, formaldehyde can be adsorbed by a cartridge made of a silica filler containing 2,4-Dinitrophenylhydrazine (2,4-DNPH) and analyzed using HPLC by referring to the measurement methods provided and recommended by the WHO, NIOSH, and others [1,2,3,4,5].

Alkylamines that can provide electrons to the carbonyl carbon, such as 2,4-DNPH, have recently been studied as an adsorbent material for formaldehyde adsorption. Since the alkylamine itself has a viscous liquid form, it is difficult to use it as the adsorbent. Therefore, supporting substrates such as activated carbon, zeolites, MOFs, or porous silicas have been used to hold it [6,7,8,9,10,11,12,13]. There are three general methods for using alkylamine or introducing the amino functional group to these substrates:

1. Amine impregnation method: directly introduces alkylamine into the prepared substrate’s pore or structure [14,15,16,17].
2. Grafting method: makes chemical bonds between the prepared substrate and the introduced amino functional groups [18,19].
3. Co-condensation method: one-pot synthesis of amino functional group-substrate [20,21,22].

Among the three methods, the amine impregnation method shows the highest adsorption ability by introducing a large number of alkylamines into the substrate. However, the amount of impregnated alkylamine differs according to the substrate and much research is still needed for recycling. Although the grafting method is advantageous for reusing the adsorbent, the amount of introduced amine is quite limited. The co-condensation method is known for the short one-pot synthesis process and also can be recycled. However, it is hard to regulate the pH when adding an amine functional group during the synthesis and it is difficult to control the morphological particle shape as intended [23,24]. Therefore, in this work, we carried out experiments for the adsorption of formaldehyde using TEPA through the amine impregnation method, which demonstrated excellent performance.

To support and contain alkylamines, the various materials mentioned above can be considered as substrates. Among them, research on silica materials with excellent cost-effective performance has rapidly increased [25,26,27]. In particular, mesoporous silica, conventionally well-known silica such as MCM-41, MCM-48, and SBA-15, and other silicas with unique structures have been studied and used as substrates for a long time [28,29,30,31]. These conventional silicas usually show outstanding surface area and highly ordered pores but a low pore volume. Even in the case of hollow silica, which is known for having a high pore volume, the synthesis procedure is complex, and the utilization of hollow internal pores is challenging. Due to the existing weaknesses in using conventional silicas as support substrates, there was demand for a more straightforward synthesis process and a higher pore volume with larger pore size. To address these issues, dendritic mesoporous silica was synthesized and tested as a substrate, anticipating improved performance with high pore accessibility, owing to its three-dimensional pore structure and excellent pore volume. Dendritic mesoporous silica and the widely used conventional silica SBA-15 (Santa Barbara Amorphous-15) were synthesized, and their formaldehyde adsorption abilities were compared using the amine impregnation method.

## 2. Materials and Methods

### 2.1. Materials

Tetraethylorthosilicate (TEOS, 98 wt%, Sigma-Aldrich-Korea, Seoul, Republic of Korea) was used as a silica precursor, H_2_O was used as both solvent and hydrolysis agent, Hexadecyltrimethylammonium p-toluenesulfonate (CTATos, 98%, Sigma-Aldrich-Korea, Seoul, Republic of Korea) was used as a template, and Triethylamine (TEA, 99%, DAEJUNG, Siheung-si, Republic of Korea) was used as a catalyst for the synthesis of DMS. For the synthesis of SBA-15, TEOS and H_2_O were also used for the same purpose. Poly (ethylene glycol)-block-poly (propylene glycol)-block-poly (ethylene glycol) (PEG-PPG-PEG; Pluronic P123, Sigma-Aldrich-Korea, Seoul, Republic of Korea) was used as a template, and chloric acid (HCl, 35–37 wt%, Samcheon, Seoul, Republic of Korea) was used as a catalyst. Absolute ethyl alcohol (EtOH, 99.9%, DAEJUNG, Siheung-si, Republic of Korea) was used as a solvent and utilized to remove the surfactant. Tetraethylene pentaamine (TEPA, 93%, Kanto Chemical, Tokyo, Japan) was used as an alkylamine and ethanol was used as both solvent for the impregnation method and solution for washing surfactants from the synthesized DMS and SBA-15. Acetonitrile (ACN, 99.9%, Sigma-Aldrich-Korea, Seoul, Republic of Korea) was used as a mobile phase and elution solvent for the HPLC analysis.

### 2.2. Dendritic Mesoporous Silica (DMS)

A mixture of 100 mL of H_2_O, 2.04 g of CTATos, and 0.235 g of TEA was put in a 3-neck round flask and stirred for 30 min at 80 °C, 500 rpm. As a precursor, 14.56 g of TEOS was added and then stirred for 2 h at 80 °C, 500 rpm. The silica slurry obtained by the above method was washed and filtered with EtOH several times. Then the white wet solids were dried in an oven at 100 °C for 2 h. After grinding using a mortar, the white powder was calcined at 550 °C for 4 h to remove residual organics. White and slightly transparent powder was obtained [32,33,34].

### 2.3. SBA-15 (Santa Barbara Amorphous, SBA-15)

SBA-15 was made by referring to some research [35,36,37]. After 6 g of P123 was well dissolved in the mixture of 164.5 g of H_2_O, 30 g of HCl was added and then stirred for 1 h at 40 °C, 500 rpm. As a precursor, 12.68 g of TEOS was quickly added dropwise and then stirred at 40 °C for 20 h, 500 rpm. Then, at 90 °C without stirring, it was aged for 36 h. The silica slurry obtained by the above method was washed and filtered with EtOH and H_2_O several times. Then the white wet solids were dried in an oven at 100 °C for 2 h. After grinding using a mortar, the white powder was calcined at 550 °C for 6 h to remove residual organics. White powder was obtained.

### 2.4. Amine Impregnation Method

As a substrate, 1 g of silica powder (DMS and SBA-15) was dispersed in 100 mL of EtOH and then stirred at 30 °C for 30 min at 900 rpm. The fixed capacity of TEPA (0.5 g, 1.5 g, 2.5 g) was added and then stirred at 30 °C for 1 h at 700 rpm. Then, the solvent was removed using a rotary evaporator at 50 °C until only the powder remained. The obtained powder was dried in a convection dryer oven at 100 °C for 1 h to remove the residual EtOH. As the amount of impregnated TEPA was increased, the obtained powder was more yellow and clumped than the less impregnated powder [14]. The amine-impregnated DMS was labeled DAI and the amine-impregnated SBA-15 as SAI. The amount of impregnated TEPA was indicated after ‘-‘. Each substrate and the amount of impregnated amine of adsorbent are summarized in Table 1. As SBA-15 has a lower pore volume than DMS, 2.5 g of TEPA-impregnated SBA-15 could not be obtained as a powder, and because the powder texture was required for the formaldehyde adsorption, the sample ‘SBA-15-Amine Impregnated-2.5 g (SAI-2.5 g) was excluded.

### 2.5. Formaldehyde Adsorption

**Figure 1:**
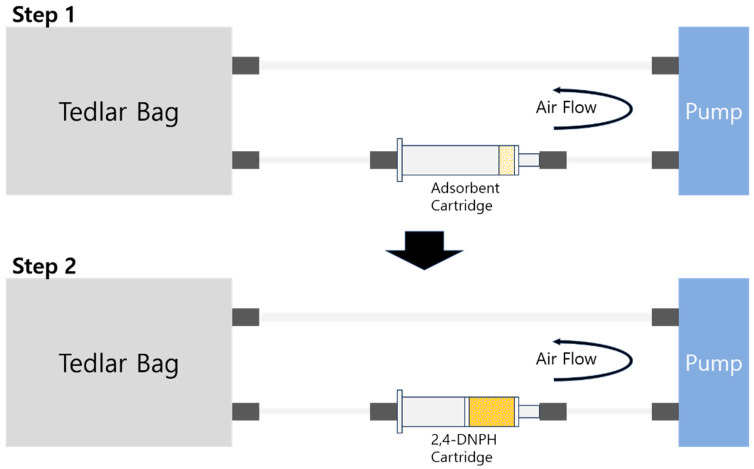
Schematic figure of formaldehyde adsorption steps.

The fixed weight of prepared adsorbent (TEPA + substrate) was placed in a polypropylene tube to make an adsorbent cartridge with 0.02 g of cotton scrap to prevent leaking of the powder while pumping the air. The fixed concentration and amount of formaldehyde solution was injected into a 3 L Tedlar bag using a microsyringe and then the bag was filled with N_2_ gas. The Tedlar bag filled with formaldehyde and N_2_ gas was put in convection dryer oven at 70 °C for 1 h to ensure the formaldehyde solution was fully gasified. In the sequence of the gasified formaldehyde Tedlar bag, adsorbent-filled cartridge, and air pump, and back to the Tedlar bag, they were connected and the air was then pumped at a flow rate of 0.5 L/min for 18 min. The labels were marked with an ‘F-‘ in front of the adsorbents’ name after formaldehyde adsorption. To calculate the amount of remaining formaldehyde, the adsorbent cartridge was replaced with a 2,4-DNPH cartridge and then the air was pumped at a flow rate of 0.5 L/min for another 18 min. The used 2,4-DNPH cartridge was eluted with 5 mL of acetonitrile for HPLC analysis. Figure 1 shows the detailed procedure of the adsorption.

### 2.6. Characterization

The textural and structural properties of DMS and SBA-15 were analyzed using a Scanning Electron Microscope (SEM, JSM-IT500LA, JEOL Ltd., Tokyo, Japan), a Field Emission-Scanning Electron Microscope (FE-SEM, S-4800, Hitachi Ltd., Tokyo, Japan), and a Field Emission-Transmission Electron Microscope (FE-TEM, HF-3300, Hitachi Ltd., Tokyo, Japan). An X-ray diffractometer (XRD, Empyrean, Malvern Panalytical Ltd., Worcestershire, UK) was used to investigate and support the structural properties of the synthesized silica. The XRD was carried out in 2 θ scan Gonio mode with Cu-Kα rays at both a low angle (0.5–5°, 0.05 of step size, 7 s per step) and a wide angle (5–80°, 0.05 of step size, 1 s per step). Surface and pore properties were analyzed using the N_2_-adsorption-desorption isotherm. The surface area was calculated using the Brunauer–Emmett–Teller (BET) method. The pore size distribution and pore volume were estimated using the non-local density functional theory (NLDFT) method and the Barrett–Jyner–Halenda (BJH) method. Through FT-IR (Cary 630, Agilent Inc., Santa Clara, CA, USA), the impregnated TEPA and the captured formaldehyde were confirmed. Using HPLC (Agilent 1260 Infinity & Agilent 6420 Triple Quad LC/MS/MS, Agilent Inc., Santa Clara, CA, USA), the amount of adsorbed formaldehyde was calculated by subtracting the amount of formaldehyde-2,4-DNPH derivative from the fixed amount of formaldehyde in the Tedlar bag. A C18 column (3.0 × 50 mm, 2.7 µm, Poroshell 120 EC-C18, Agilent Inc., Santa Clara, CA, USA) was used. The mobile phase ratio was (A) water and (B) acetonitrile (A:B = 60:40, v/v), the flow rate was 0.4 mL/min, and the running time was 5 min. Using UV/DAD, the formaldehyde-2,4-DNPH derivative was detected at the wavelength of 360 nm.

## 3. Results and Discussion

**Figure 2:**
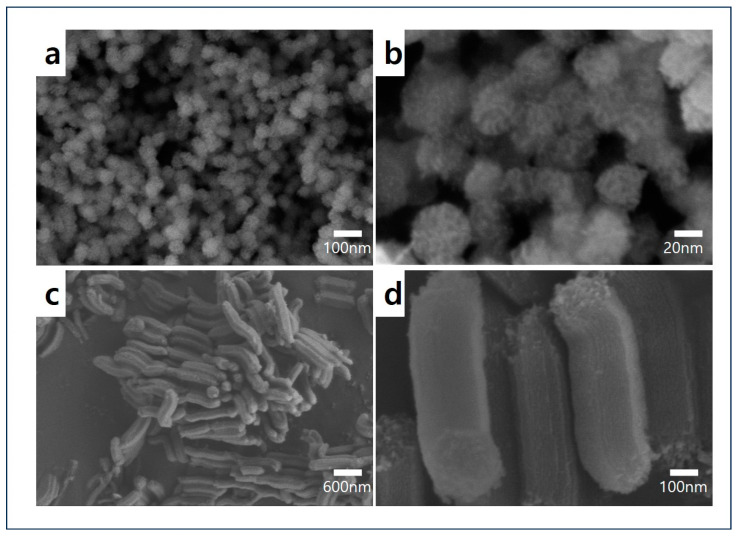
SEM images before and after magnification: (**a**,**b**) DMS and (**c**,**d**) SBA-15.

**Figure 3:**
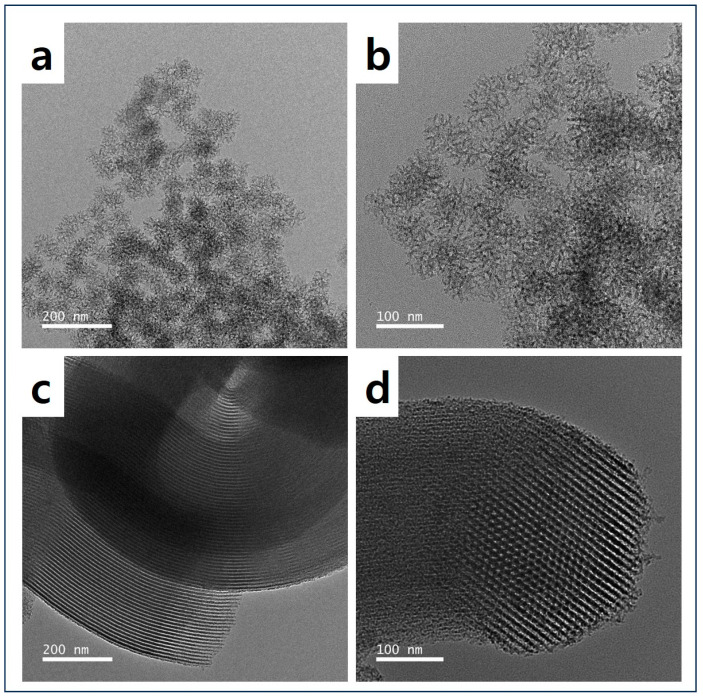
TEM images before and after magnification: (**a**,**b**) DMS and (**c**,**d**) SBA-15.

To confirm the structure and texture of DMS and SBA-15, the resulting images from SEM and TEM were used. Figure 2a,b show the successful synthesis of DMS with its unique pore texture, while Figure 3a,b demonstrate its hierarchical dendritic shape with a radial pore structure [33]. The rod-like particle and one-way cylindrical pore structure of SBA-15 were confirmed, as shown in Figure 2c,d and Figure 3c,d [38,39,40]. Therefore, it can be assumed that DMS has various pore sizes and high pore accessibility, whereas SBA-15 appears to have a two-dimensional (2D) hexagonal pore structure, making it challenging for TEPA to access the center of the pores.

**Figure 4:**
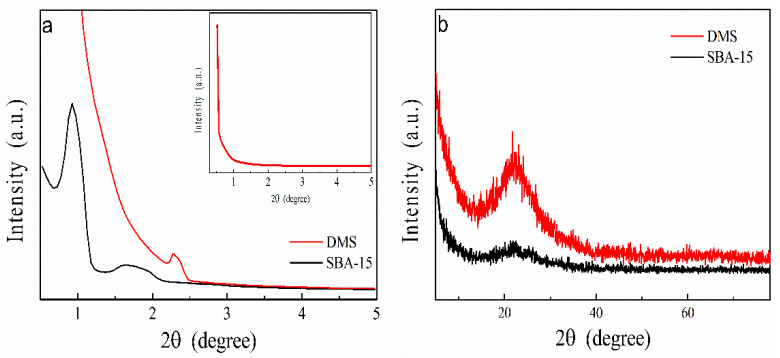
XRD patterns of DMS and SBA-15: (**a**) low-angle XRD and (**b**) wide-angle XRD.

XRD data support their structural properties. In the low-angle XRD data in Figure 4a, SBA-15 reveals three typical hexagonal (P6m) mesoporous structure peaks, including strong (100), weak (110), and (200) Bragg reflections [37,38]. Even though both DMS and SBA-15 have a disordered atom arrangement, the peaks indicating ordered as structure are shown only in SBA-15 since the pores in SBA-15 are arranged in a hexagonal tiling. DMS shows only one small peak at 2.3°~2.4° which means that it has a smaller mesopore structure compared to SBA-15 [41,42,43,44,45]. Since the surfactant P123 has a longer chain than CTATos, the micelle of DMS will be smaller than that of SBA-15. Therefore, the interpretation that the pores made by the micelle in DMS are smaller than the pores in SBA-15 is reasonable. In the wide-angle XRD data shown in Figure 4b, the broad peak at 20°~25° indicates that both DMS and SBA-15 have amorphous characteristics like any other porous silica [15,46]. However, it seems that these small pores are incompatible with the high pore accessibility we were expecting.

**Figure 5:**
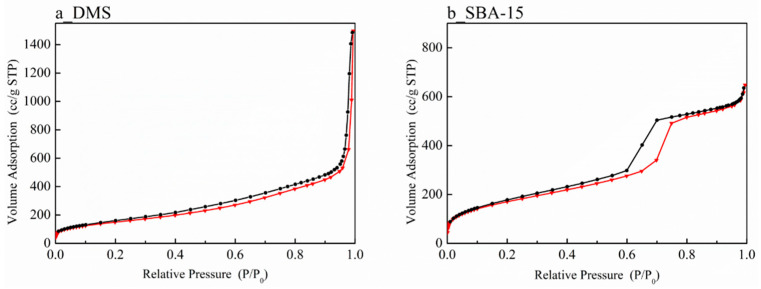
N_2_-physisortion isotherm: (**a**) DMS and (**b**) SBA-15.

**Figure 6:**
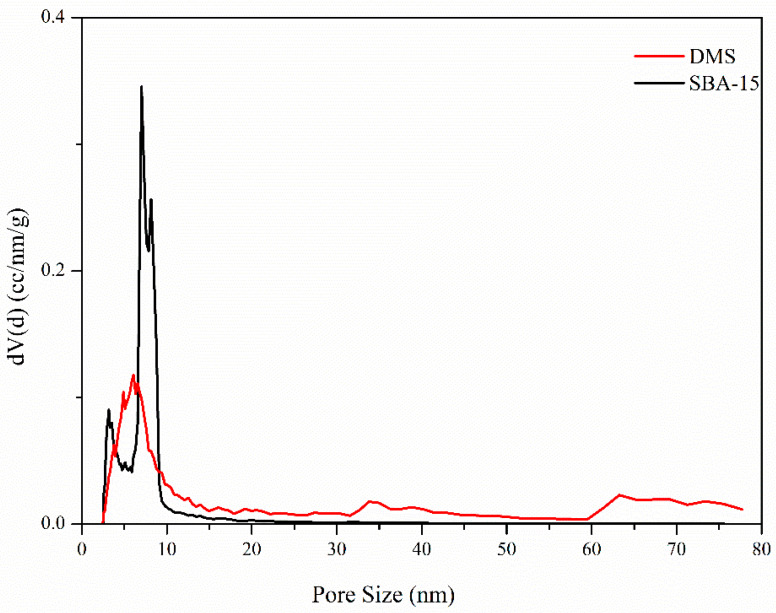
Pore size distribution of DMS (red line) and SBA-15 (black line) obtained using the NLDFT method.

N_2_-physisorption isotherms and the pore size distribution of DMS and SBA-15 in Figure 5 and Figure 6 help to solve this issue. In the case of SBA-15 (Figure 5b), when accompanied by capillary condensation around the relative pressure range of 0.6–0.8 (P/P_0_), it exhibits a typical Type IV(a) characteristic according to the IUPAC classification, indicating cylindrical pores with a width wider than 4 nm. On the other hand, the isotherm of DMS (Figure 5a) looks like a mixture of Type II and IV with a broad pore size distribution area [15]. The part of the Type IV isotherm hysteresis loop indicates the presence of mesopores in a broad area. The isotherm of Type II suggests the possibility of the existence of macropores. Therefore, it can be assumed that DMS has pores ranging in size from meso to macro. Since the volume adsorption at the relative pressure of 0.3 (P/P_0_) of SBA-15 is larger than that of DMS, it is able to be estimated that SBA-15 has a slightly larger surface area. However, with a larger scale of the *Y*-axis, the wider area of the hysteresis loop indicates that the actual pore volume of DMS will be higher than that of SBA-15 [47].

Figure 6 shows the pore size distributions of DMS and SBA-15. While the pores of SBA-15 are concentrated in the 7–8 nm range, the pores of DMS cover almost the entire mesopore area, ranging from several nanometers to dozens of nanometers. In particular, there is a definite concentration of pores in the 5–7 nm range, albeit fewer than in SBA-15, supporting the low-angle XRD data which indicated the existence of smaller pores. Although the mean particle size of DMS is about 60 nm, because of the aggregation of particles, the sums of its neighboring pores and the vacancies among particles appear unrealistically larger than its actual particle size. Additionally, the open-ended radial pore structure of DMS may be a reason for this result, as an opened pore can be recognized as a part of the wall of a larger pore [48]. These findings are also consistent with the morphological features of the particles observed in SEM and TEM images. The calculated and summarized pore properties are shown in Table 2.

**Figure 7:**
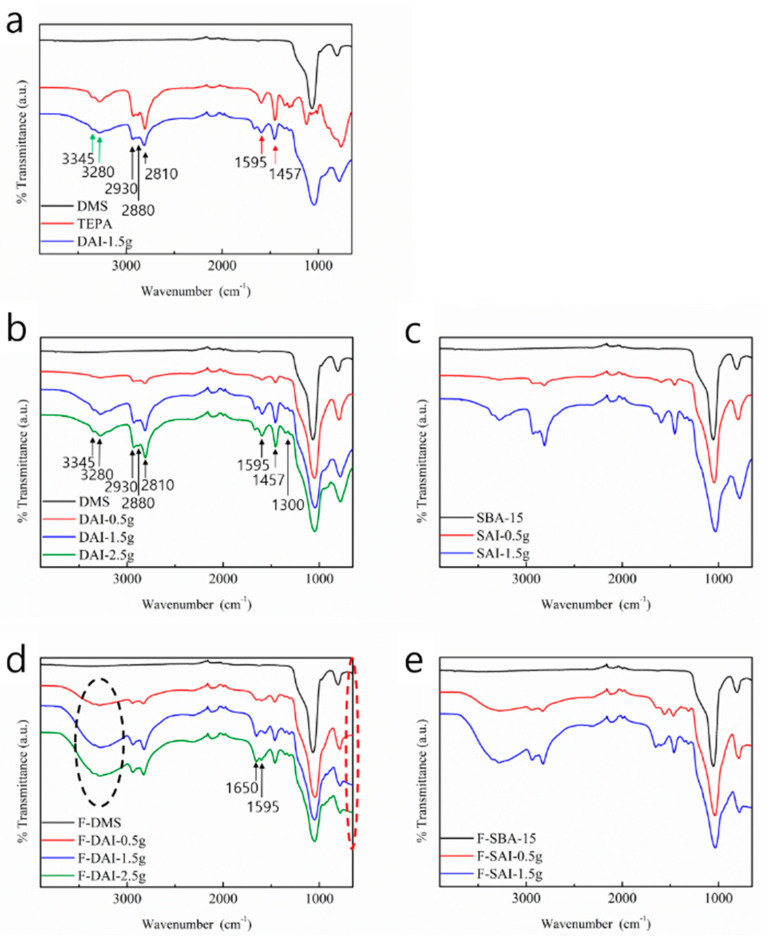
FT-IR spectra before and after TEPA impregnation and formaldehyde adsorption. (**a**) Pure substrate, TEPA, and DAI-0.5 g. (**a**–**c**) N–H bending (1595 cm^−1^), C–H bending (1457 cm^−1^), C–H stretching (2810 cm^−1^, 2880 cm^−1^, and 2930 cm^−1^) and N–H stretching (3280 cm^−1^ and 3345 cm^−1^) peaks of the impregnated TEPA in DMS and SBA-15 are shown. (**d**,**e**) N–H wagging (under 750 cm^−1^), C=N stretching (1650 cm^−1^), and broad O-H stretching (3280 cm^−1^ centered) peaks.

After confirming the characteristics of DMS and SBA-15 through the analyses above, TEPA was introduced into the DMS and SBA-15. Since alkylamines such as TEPA can capture the gas state formaldehyde by chemical bonding, we anticipated the adsorbents could adsorb the formaldehyde after the TEPA was introduced. Before and after TEPA impregnation and formaldehyde adsorption, the adsorbents were analyzed using FT-IR and then the process of adsorption was determined. The FT-IR spectra in Figure 7 shows TEPA was successfully introduced before and after the formaldehyde adsorption. In Figure 7a, DMS, TEPA, and TEPA-impregnated DMS (DAI) were compared. Two peaks (3280 cm^−1^ and 3345 cm^−1^) (green arrows) show the N–H stretching of primary and secondary amines. The C–H bending (1457 cm^−1^) and the N–H bending (1595 cm^−1^) peaks are shown with red arrows. One pair of symmetric and asymmetric C–H stretching (2900 cm^−1^ and 2930 cm^−1^) peaks and another C–H stretching (2815 cm^−1^) peak are shown with black arrows. None of these peaks appeared in the pure DMS but all appeared after TEPA was introduced to DMS at DAI-1.5 g. This shows silica substrate holds TEPA well as a support and the impregnation procedure was successfully carried out.

**Figure 8:**
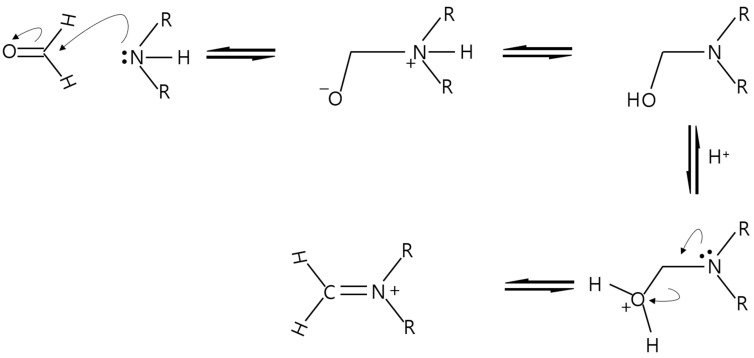
Mechanism of formaldehyde-amine reaction.

In Figure 7b,c, each peak of DMS and SBA-15 with a different amount of impregnated TEPA also shows that TEPA was successfully introduced and impregnated. Additionally, weak C-N stretching peaks (near 1300 cm^−1^) of the aliphatic amines were also confirmed. After formaldehyde adsorption, in Figure 7d,e, the broad O-H stretching (3280 cm^−1^ centered) peak was added (in the black oval area) and the imine peak (1650 cm^−1^) appeared. Below 750 cm^−1^, the N–H wag peaks increased (in the red oval area) [15,36,49,50]. These C=N and O-H peaks confirmed that formaldehyde was successfully captured by TEPA. The peak of the broad O-H stretching, which seems unrelated to formaldehyde adsorption, is one of the key pieces of evidence of the chemical reaction between formaldehyde and TEPA. After the nitrogen of TEPA gives an electron to the carbonyl carbon of formaldehyde, they form R_2_-N-CH_2_-O-H with H^+^ as an intermediate stage of the entire reaction, as illustrated in Figure 8.

**Figure 9:**
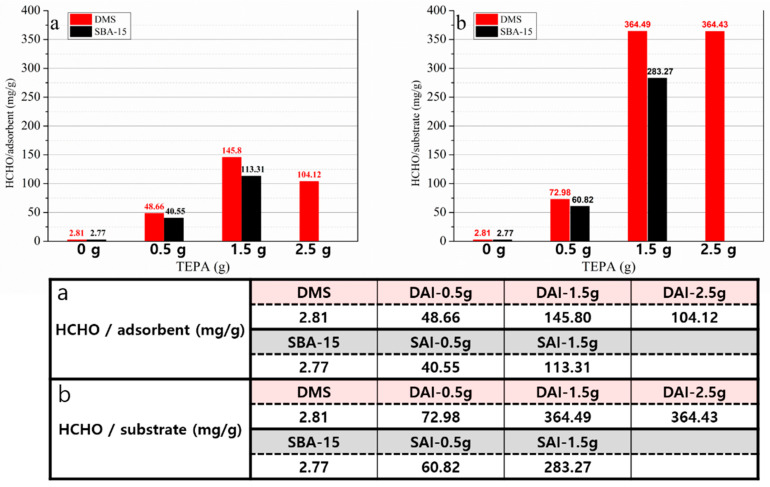
Calculated adsorbed formaldehyde using HPLC analysis: (**a**) the amount of captured formaldehyde (mg) per 1 g of adsorbent (TEPA + substrate); (**b**) the amount of captured formaldehyde (mg) per 1 g of substrate.

Since formaldehyde is a very small molecule, it is challenging to directly detect and calculate its amount. However, using the 2,4-DNPH cartridge, the formed formaldehyde-2,4-DNPH derivative can be detected and quantified by HPLC [1,2,3]. Since one 2,4-DNPH molecule reacts with one formaldehyde molecule, via detection of the derivative calculating the amount is straightforward. Under the recommended flow rate condition of less than 0.5 L/min, the adsorption of formaldehyde was carried out by circulating the air in the Tedlar bag at a flow rate of 0.5 L/min for 18 min using a 3 L Tedlar bag filled with a fixed concentration of formaldehyde and nitrogen gas. After being captured by the prepared TEPA-impregnated adsorbent, the used adsorbent-filled cartridge was replaced with a 2,4-DNPH cartridge, and then the remaining formaldehyde could be captured by the 2,4-DNPH cartridge. Since the formaldehyde captured by 2,4-DNPH forms a formaldehyde-2,4-DNPH derivative in a one-to-one correspondence, the formaldehyde captured by the adsorbent was calculated by subtracting the amount of formaldehyde-2,4-DNPH derivative from the total amount of formaldehyde in the Tedlar bag at the start of the experiment. Through HPLC analysis, the formaldehyde-2,4-DNPH derivative eluted by acetonitrile was detected and its concentration was calculated. Figure 9 presents the calculated results of the HPLC analysis.

As more TEPA was impregnated, the adsorption of formaldehyde per weight of the amine-impregnated adsorbent increased. However, in DAI-2.5 g, when 2.5 g of TEPA was introduced, the amount of formaldehyde adsorbed decreased. The amount of adsorbed formaldehyde per weight of substrate did not decrease but also did not increase. Compared to SAI, DAI adsorbed more formaldehyde at every point with the same amount of impregnated TEPA, and this was the same both per substrate and per whole adsorbent. This result is consistent with other research, suggesting that the pores of the substrate can become blocked when TEPA is impregnated into the pores. As TEPA fills the pores, the difference in the pore size and pore volume of the substrate results in varied pore availability, as expected [15,17,48].

## 4. Conclusions

Dendritic mesoporous silica (DMS) and Santa Barbara Amorphous-15 (SBA-15), which have different pore characteristics, were prepared and their abilities as adsorbents were compared. Focusing on the pore volume and pore structure, the surface area of DMS and SBA-15 was adjusted to similar values. Among the various methods, the amine impregnation method was introduced because of its simple preparation sequences and high adsorption performance. Through HPLC, the amount of adsorbed formaldehyde was calculated by subtracting the amount of formaldehyde-2,4-DNPH derivative from the total amount of formaldehyde. When alkylamine (TEPA) was introduced and impregnated into the porous substrate, the maximum amount of impregnated amine differed based on the pore volume. The sample in which excessive amine was introduced was excluded because a powder texture was required for use as the adsorbent. When the same amount of TEPA was impregnated, DMS, which has larger pore volume and pore size, adsorbed more formaldehyde. As the impregnated TEPA increases, it blocks the pores of the substrates, reducing the pore accessibility and utilization. In the case of SBA-15, because of its cylindrical two-dimensional pore structure, the whole pore or the inner space cannot be utilized when the impregnated TEPA starts to block the end of the pore. Alternatively, in the case of DMS, the pore blocking affects the pore utility less than in the case of SBA-15 since DMS has a three-dimensional and radial pore structure. We now understand the significance of pore volume and pore accessibility in the amine impregnation method, and our future interest lies in finding the intersection point of particle size, surface area, pore size, and pore volume that maximizes the adsorption ability. As the three-dimensional large pore structure can accommodate various functional groups, our future focus will extend beyond the adsorption of formaldehyde to include the adsorption of other harmful VOCs found indoors.

## Figures and Tables

**Table 1:** Substrates used that impregnate TEPA and the amount of impregnated TEPA.

Adsorbent	Substrate	Amount of the Impregnated TEPA (g/1 g of Substrate)	After Impregnation (TEPA + Substrate, g)
DMS	Dendritic mesoporous silica	0	1
DAI-0.5 g		0.5	1.49
DAI-1.5 g		1.5	2.49
DAI-2.5 g		2.5	3.46
SBA-15	SBA-15	0	1
SAI-0.5 g		0.5	1.5
SAI-1.5		1.5	2.47

**Table 2:** Surface and pore properties of DMS and SBA-15.

	Multipoint BET (m^2^/g)	Total Pore Volume (cc/g)	Average Pore Diameter (nm)
DMS	544.90	2.23	17.00
SBA-15	624.98	0.91	6.41

## Data Availability

Data is contained within the article.
